# Cottonseed Meal Protein Hydrolysate Improves the Growth Performance of Chinese Mitten Crab (*Eriocheir sinensis*) by Promoting the Muscle Growth and Molting Performance

**DOI:** 10.1155/2023/8347921

**Published:** 2023-06-28

**Authors:** Chao-Fan He, Wen-Bin Liu, Ling Zhang, Wei-Liang Chen, Zi-Shang Liu, Xiang-Fei Li

**Affiliations:** Key Laboratory of Aquatic Nutrition and Feed Science of Jiangsu Province, College of Animal Science and Technology, Nanjing Agricultural University, No. 1 Weigang Road, Nanjing 210095, Jiangsu, China

## Abstract

Growth retardation and prolonged marketing cycle have been noticed in the practical aquaculture of Chinese mitten crab (*Eriocheir sinensis*) fed with artificial feed. Plant protein hydrolysates contain a large number of small peptides and free amino acids, which can improve the growth performance of aquatic animals. However, the potential mechanisms are still not well elucidated. In this research, the influences of cottonseed meal protein hydrolysate (CPH) on the growth, feed utilization, muscle growth, and molting performance were investigated in *E. sinensis*. A total of 240 crabs (mean body weight 37.32 ± 0.38 g) were individually randomly distributed to six diets supplemented with 0%, 0.2%, 0.4%, 0.8%, 1.6%, and 3.2% of CPH for 12 weeks. These findings indicated that the addition of CPH at 0.4% significantly increased the survival rate, body protein gain, apparent protein utilization, trypsin and pepsin activities, and the methyl farnesoate content. When the dose reached 0.8%, the weight growth ratio, meat yield, ecdysone concentration, and the transcription of the ecdysteroid receptor all significantly increased, while the transcriptions of both myostatin and molt-inhibiting hormone significantly decreased. When CPH was added at 1.6%–3.2%, the feed conversion ratio, body crude protein content, Na+/K+-ATPase activity, and the molting ratio were all significantly improved, while the opposite was true for the transcription of the transforming growth factor-*β* type I receptor. The investigation results indicated that when added above 0.4%, CPH could stimulate the growth performance of *E. sinensis* and promote the muscle growth and molting performance.

## 1. Introduction

As an excellent species for aquaculture, Chinese mitten crab (*Eriocheir sinensis*) has attracted considerable attention in China with an annual production of 790,000 tons [1]. To date, trash fish is still the main bait for this species in practical aquaculture, as it eventually leads to an increasing shortage of the trash fish resource and high farming cost [2]. In addition, the sources and species of trash fish are variable [3], making it difficult to standardize the crab farming. Furthermore, feeding large amounts of trash fish into artificial ponds would easily deteriorate water quality, as they inevitably result in a high susceptibility to disease outbreaks [4], since trash fish is perishable [5]. Comparatively, artificial feed has the benefits of stable product quality, balanced nutrition, storage ease, and cheap price, and has been gradually accepted by crab farmers. However, some shortcomings have also gradually emerged during the application of aquafeed, mainly a slow growth rate and the prolonged market cycle. Actually, the Chinese consumers are fond to buy *E*. *sinensis* during the Chinese Mid-Autumn Festival and the Chinese National Day holidays (from September to October), during which *E. sinensis* has a high unit price. However, at this time, crabs-fed artificial feed are not yet fully grown and usually have poor muscle fullness. This prevents them from capturing the market in time for peak prices, ending up with lower prices and economic benefits compared with the trash fish-fed ones. This has severely limited the promotion and implementation of artificial feed for crab farming.

As an arthropod, the growth of *E. sinensis* mainly depends on continuous molting [6], which is in turn closely related to the muscle growth. Generally, *E. sinensis* needs to initiate the molting activity to move to the next growth stage, when muscle grows to a certain fullness during the intermolt period [7], as it explains its discontinuous weight gain (WG). Accordingly, *E. sinensis* is able to gain weight not only through the growth, proliferation, and differentiation of muscle cells, but also through the consequent molting activities brought about by the muscle growth. Therefore, promoting the muscle growth and molting activity might be a key in resolving the problems of growth retardation, prolonged market cycle, and reduced economic efficiency in crab farming.

Plant protein sources have been widely accepted as a substitute for fish meal in aquafeeds due to their high quality and low cost. However, high incorporations of plant proteins into feed negatively affect the growth performance of aquatic animals owing to the imbalance in amino acid and high level of antinutritional factors [8]. Recently, the enzymatic hydrolysis technology has been used to treat plant proteins. Indeed, recent researches have demonstrated that hydrolysis markedly enhances the content in small peptides and free amino acids, both of which can enhance the protein deposition and growth performance of animals [9–11]. For example, in our previous study, the nutritional compositions of several plant protein (including soybean meal, cottonseed meal, rapeseed meal, and peanut meal) hydrolysates were analyzed with the cottonseed meal protein hydrolysate (CPH) and shown the best value [12]. Consequently, several researches have been carried out to evaluate the growth and immune-stimulating effects of CPH in fishes such as *Carassius auratus gibelio* [10], *Cyprinus carpio* var. Jian [13], and *Megalobrama amblycephala* [14]. In crustaceans, CPH has been evaluated as an appetite stimulant as applied to the *E. sinensis* with its effects on feed ingestion speed, antioxidant capacity, and immunity investigated [15]. Nevertheless, the potential mechanics of these beneficial influences are yet to be well interpreted.

Bearing this in view, this present research was carried out to investigate the influences of diet CPH levels on growth, feed utilization, the muscle growth, and molting performance of *E. sinensis*. The objective is to find an effective nutritional intervention to resolve the problems of growth retardation, prolonged market cycle, and reduced economic benefits in this species-fed artificial feed, thereby ultimately contributing to the use of aquafeed in crab farming.

## 2. Materials and Methods

### 2.1. Animal Ethics

All animal treatments were authorized by the Animal Care and Use Committee in Nanjing Agricultural University (Nanjing, China) (ethics number: SYXK (Su) 2011 0036).

### 2.2. CPH and Diets

CPH was prepared according to the method outlined by Xia et al. [16]. The protein profiles of CPH, including the contents of protein (1,893–21,828 Da), soluble protein, amino acid (74–180 Da), and small peptide (180–1,983 Da), were measured previously in our lab [10, 17, 18] and were summarized in Table 1. The experimental diets were complemented with 0%, 0.2%, 0.4%, 0.8%, 1.6%, and 3.2% of CPH at the compensation of cottonseed meal added to the basal diet with starch adopted to maintain the diets isonitrogenous. The diets were labeled as CPH0, CPH0.2, CPH0.4, CPH0.8, CPH1.6, and CPH3.2. The diet formulations are detailed in Table 2. Soybean meal, cotton meal, rapeseed meal, peanut meal, fish meal, and blood meal were incorporated as protein sources. Both fish oil and soybean oil were included as lipid sources, and *α*-starch was adopted as the main carbohydrate source. All feed materials were crushed by an 80 mesh grinder and then mixed thoroughly. The lipid sources were added subsequently with the feed materials thoroughly mixed again. Finally, distilled water (30% of the raw material weight) was added and mixed thoroughly with other feed stuffs. Then, the mixed raw materials were extruded through a single-screw grinder with a die diameter of 2.0 mm and were air-dried and cut into suitable sizes (about 1.8 cm long) for storage at −20°C.

### 2.3. Crab Management

*E*. *sinensis* was caught at the same time in the same pond for the experiment. Specifically, crabs in the intermolt stage were harvested within 2 days after the peak of the last molting. The ones showed different molting stages were removed according to the methods reported by Diez and Lovrich [19]. After the harvest, crabs were temporarily cultured in several concrete ponds for 1 week by providing basal diet twice a day. Then, 240 individuals (mean initial weight: 37.32 ± 0.38 g) and vigor were randomly picked and transferred into 24 cement ponds (0.5 × 0.5 × 0.8 m in length, width, and height, respectively). Each pond held 10 crabs in a male-to-female ratio of 1 : 1. Then, the experimental diets were randomly fed to the crabs with each tested in four ponds. To avoid cannibalism, crabs at the later premolting stage were transferred to the cement pond with the same environment for separate rearing. The feeding trial lasted for 12 weeks with the number of crab shells counted daily within each pond. In this period, water temperature, dissolved oxygen, and pH were maintained at 24–28°C, 5.1 mg/L, and 8.0–8.5, respectively.

### 2.4. Sample Collection and Analysis

#### 2.4.1. Sample Collection

Eight crabs were randomly kept as the initial sample at the commencement of the culture trial. At the termination of the feeding period, all crabs were starved for 24 hr and then subjected to hypothermia anesthesia by using ice bags in order to reduce their activity. Each crab was weighed afterward in order to calculate growth data. Two crabs were randomly picked from every pond for sampling, which was performed on the ice bag. First, hemolymph was collected using sterile syringes filled with anticoagulant [20]. The hemolymph was centrifuged at 3,500 rpm at 4°C for 20 min with the supernatants were collected and stored at −20°C for future analysis. Then, the whole hepatopancreas was collected and stored at −80°C for future use. Finally, the claw, leg, and abdomen muscle and the triangular membrane were all removed with scalpels and forceps and were weighted and stored at −80°C for subsequent analysis.

#### 2.4.2. Proximate Composition Analysis

According to the official AOAC method [21], the contents of moisture, crude protein, crude fat, and crude ash were determined. Briefly, the moisture content was determined by calculating the weight loss of the samples dried at 105°C to a constant weight. The contents of crude protein, crude lipid, and crude ash were determined by using the Kjeldahl system, Soxhlet extractor, and muffle furnace (at 550°C for 4–6 hr), respectively.

#### 2.4.3. Measurement of Amino Acid Profile

The muscle samples were vacuum-sealed in ampoules with 6 N of HCl at 110°C for 24 hr, and then the HCl was blown away by nitrogen gas. The samples were then redissolved with 0.1 N of HCl with the supernatant filtered through a 0.22 *μ*m filter into the amino acid assay tubes. The amino acid profile was determined using an automated amino acid detector (L-8900; Hitachi High-Technologies, Inc., Tokyo, Japan).

#### 2.4.4. Determination of the Activities of Digestion-Related Enzymes

Hepatopancreas was weighed and homogenized with a normal saline (dilution of 1 : 10). The homogenates were then centrifuged at 5,000 rpm at 4°C for 10 min. The supernatants were assayed for protein concentration using bovine serum albumin (Sigma, USA) to calculate the enzyme activity. Then, the activities of trypsin, pepsin, Na^+^/K^+^-ATPase, and *γ*-glutamine acyltransferase (*γ*-GT) were all determined using commercial kits produced by Jiancheng Bioenginneering Company (Nanjing, China).

#### 2.4.5. Determination of the Concentration of Molting-Related Hormones

The concentrations of methyl farnesoate (MF) and ecdysone in the hemolymph were both determined by commercial ELISA kits (Elisa Biotech, Shanghai, China) according to the manual.

#### 2.4.6. Transcriptional Analysis

The total RNA was extracted using the RNA lysis solution (Accurate Biotechnology, Hunan, China). Reverse transcription was performed using the PrimeScript reverse transcriptase (Takara), and the resulting cDNA was amplified using the SYBR Premix Extaq Kit (Takara). Reactions were performed in two steps using a thermocycler (Takara): first step at 42°C, run for 40 min; second step at 90°C, run for 2 min. Then, the samples were stored at 4°C. The Primer Premier 5.0 program was used to design the primers of the ubiquitin/ribosomal S27 fusion protein (S27, the internal reference gene), myostatin (MSTN), transforming growth factor-*β* type I receptor (EsTGFBRI), ecdysteroid receptor (EcR), retinoid X receptor (RXR), molt-inhibiting hormone (MIH), cryptocyanin (Cc), and cuticle protein cbm (CP cbm) (Table 3). The primers were synthesized by Shanghai Generay Biotech Co., Ltd., Shanghai, China. The relative expressions of these genes were all determined (MSTN in muscle, EsTGFBRI in the triangular membrane, and others in the hepatopancreas) by the 2^−*ΔΔ*CT^ method using the TaKaRa SYBR® Premix Ex Taq^TM^ II kit.

### 2.5. Statistical Analysis

The one-way analysis of variance (ANOVA) procedure of the SPSS (25.0) computer program was applied to analyze data, and the Tukey's multiple range test adopted to identify the difference among different groups. The type of significance (linear or quadratic) was then determined by using an orthogonal polynomial comparison. Unlikely, the data of total molting times were subjected by a two-way ANOVA taking into consideration the influences of culturing weeks, CPH levels, and their interaction. When data lacked normality, they were log- or inverse-transformed [26]. Data in the article were expressed as mean ± standard error. A significant difference was identified when the *P*-value was <0.05.

## 3. Results

### 3.1. Growth Performance and Muscle Composition

As listed in Tables 4 and 5, final body weight (FBW), WG, body protein gain (BPG), apparent protein utilization (APU), and muscle protein content all increased both linearly and quadratically (*P* < 0.01) with increasing CPH additions, while an opposite result was noted in feed conversion ratio (FCR) and muscle moisture content, which decreased both linearly and quadratically. The survival rate (SR) of the CPH0.4 group showed no statistical difference (*P* > 0.05) with that of the CPH1.6 group, but was significantly (*P* < 0.05) higher than those of the other treatments. Additionally, dietary CPH supplement exerted no significant effect (*P* > 0.05) on the lipid and ash content as well as the amino acid profile in muscles (Tables 5 and 6).

### 3.2. The Activities of Digestion-Related Enzymes

**Figure 1:**
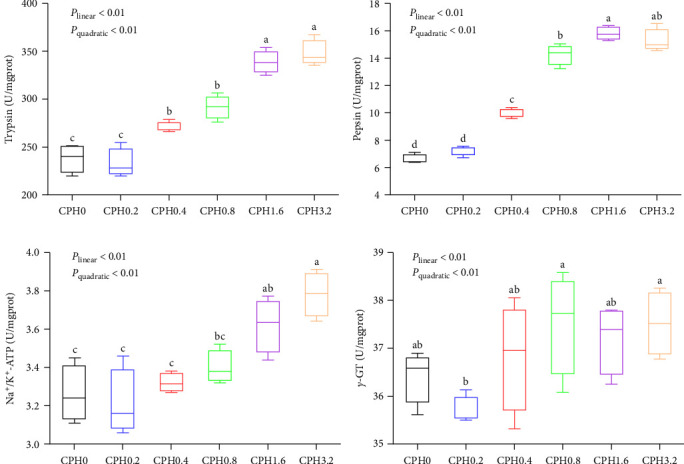
Effects of dietary cottonseed meal protein hydrolysate levels on the activities of digestion-related enzymes in the hepatopancreas of *E. sinensis*. (a) Trypsin; (b) pepsin; (c) Na^+^/K^+^-ATPase; (d) *γ*-glutamyl transpeptidase, *γ*-GT. The upper and lower limits of the box represent the first and third quartiles, while the horizontal line inside the box represents the second quartile (median). The whiskers represent the maximum and minimum values. Each data represented the mean of four replicates. The boxes assigned with different superscripts are significantly different (*P* < 0.05).

As shown in Figure 1, the activities of trypsin, pepsin, and Na^+^/K^+^-ATPase all increased both linearly and quadratically (*P* < 0.01) with increasing dietary CPH levels up to 1.6% (*P* < 0.05) and then plateaued (*P* > 0.05). In addition, the *γ*-glutamyl transpeptidase (*γ*-GT) activity of the CPH0.8 group was significantly (*P* < 0.05) higher than that of the CPH0.2 group, but showed no statistical difference (*P* > 0.05) with those of the other treatments.

### 3.3. Muscle Growth-Related Indicators

**Figure 2:**
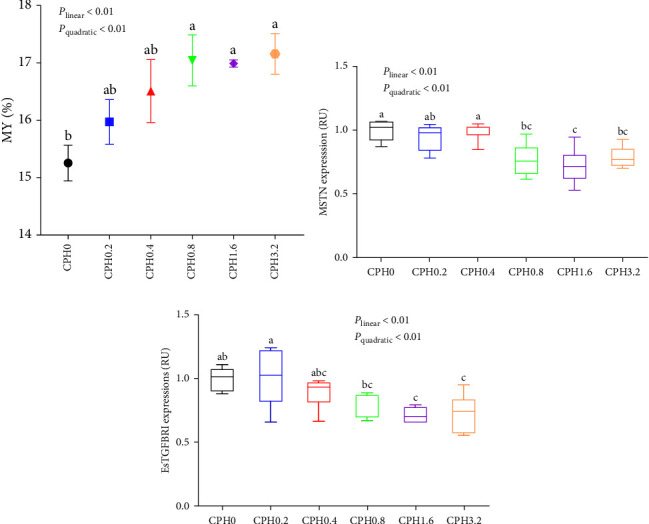
Effects of dietary cottonseed meal protein hydrolysate levels on the meat yield (MY, a) and relative expressions of myostatin (MSTN, b) and the transforming growth factor-*β* type I receptor (EsTGFBRI, c) in the hepatopancreas of *E. sinensis*. The upper and lower limits of the box represent the first and third quartiles, while the horizontal line inside the box represents the second quartile (median). The whiskers represent the maximum and minimum values. For tissue expression, data are referred to the values (relative units (RU)) found in the CPH0 group. Each data represented the mean of four replicates. Meat yield (MY) = meat weight × 100/body weight. The boxes assigned with different superscripts are significantly different (*P* < 0.05).

As shown in Figure 2, MY increased significantly both linearly and quadratically with increasing dietary CPH levels up to 0.8% (*P* < 0.01) and then plateaued (*P* > 0.05). The transcriptions of MSTN and EsTGFBRI both decreased linearly and quadratically (*P* < 0.01), when CPH supplement reached 0.8% and 1.6%, respectively, and then plateaued (*P* > 0.05) (Figure 2).

### 3.4. Molting-Related Indicators

**Figure 3:**
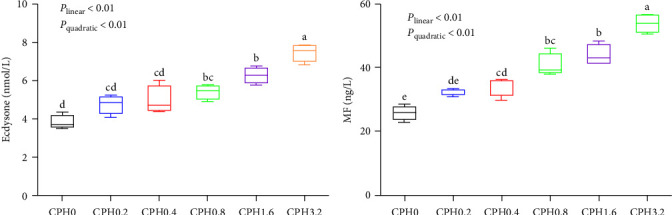
Effects of dietary cottonseed meal protein hydrolysate levels on the concentration of molt-related hormones in the hemolymph of *E. sinensis*. (a) Ecdysone; (b) methyl farnesoate, MF. The upper and lower limits of the box represent the first and third quartiles, while the horizontal line inside the box represents the second quartile (median). The whiskers represent the maximum and minimum values. Each data represented the mean of four replicates. The boxes assigned with different superscripts are significantly different (*P* < 0.05).

As shown in Figure 3, the concentrations of ecdysteroid and MF both increased linearly and quadratically (*P* < 0.01) with increasing dietary CPH levels.

**Figure 4:**
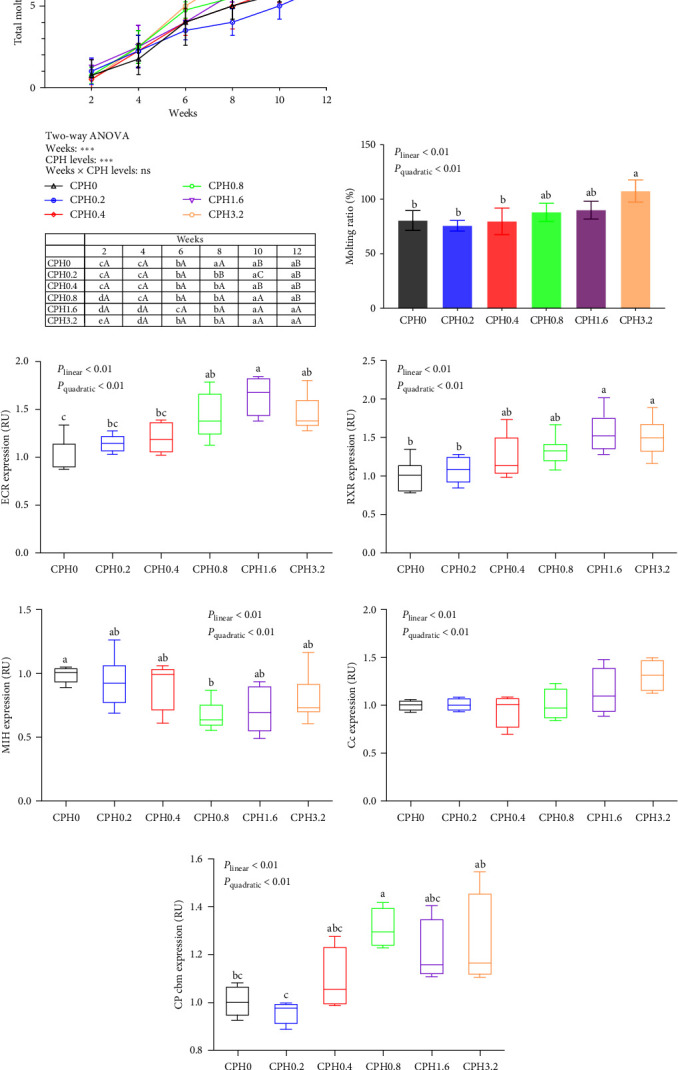
Effects of dietary cottonseed meal protein hydrolysate levels on (a) total molting times and (b) moting ratio (molting ratio (%) = 2 × the number of molting/(final crabs number + initial crabs number) as well as the relative expressions of (c) ecdysteroid receptor (EcR), (d) retinoid X receptor (RXR), (e) molt-inhibiting hormone (MIH), (f) cryptocyanin (Cc), and (g) cuticle protein cbm (CP cbm). The upper and lower limits of the box represent the first and third quartiles, while the horizontal line inside the box represents the second quartile (median). The whiskers represent the maximum and minimum values. For tissue expression, data are referred to the values (relative units (RU)) found in the CPH0 group. Each data represented the mean of four replicates. The boxes assigned with different superscripts are significantly different (*P* < 0.05). Different lowercase letters indicate significant differences (*P* < 0.05) at different week points within each treatment, whereas different capital letters indicate significant differences (*P* < 0.05) among six treatments at each sampling point. ns *P* > 0.05,  ^*∗*^*P* < 0.05,  ^*∗∗*^*P* < 0.01,  ^*∗∗∗*^*P* < 0.001.

As shown in Figure 4, the total molting times were significantly (*P* < 0.001) influenced by the culturing weeks and CPH levels. Meanwhile, the molting ratio increased both linearly and quadratically (*P* < 0.01) with increasing dietary CPH levels. Although the CPH supplement exerted no significant effect (*P* < 0.05) on the expression of Cc, the expressions of EcR and retinoid X receptor (RXR) both increased linearly and quadratically (*P* < 0.01) with increasing dietary CPH levels up to 1.6% (*P* < 0.01) and then plateaued (*P* > 0.05). However, the expression of MIH decreased both linearly and quadratically (*P* < 0.01) as dietary CPH doses increased from 0% to 0.8% and then plateaued (*P* > 0.05), whereas an opposite result was noted in the expression of CP cbm.

## 4. Discussion

The growth performance and body nutrient deposition of aquatic animals are mainly determined by feed quality, especially dietary nutrient composition [27]. In this study, dietary addition of CPH markedly improved the FBW, WG, SR, FCR, BPG, and APU as well as muscle protein content in *E. sinensis*, suggesting that CPH promotes the growth and body nutrient deposition of crabs. This results in expectation since the hydrolysis of plant proteins generally produces large amounts of readily absorbed hydrolysates, which could benefit the growth performance of aquatic species such as snakehead (*Channa argus*) and Chinese soft-shell turtle (*Pelodiscus sinensis*) [28, 29]. Indeed, compared with the cottonseed meal, CPH showed an increase in the content of small peptide (180–1,983 Da), free amino acid (74–180 Da), and soluble protein by 59.38%, 605.26%, and 125%, respectively. In addition, plant protein hydrolysis activates the mammalian target of rapamycin (mTOR), thereby promoting the protein deposition in fish like blunt snout bream (*M. amblycephala*) [17, 18], as it may also help explain the increased muscle crude protein content of *E. sinensis* subjected to CPH intervention in this study.

The growth performance of animals is highly correlated with their digestion capacity [30], which could be indicative of the activities of digestive enzymes [31]. In this research, CPH supplementation significantly increased the activities of trypsin, pepsin, Na^+^/K^+^-ATPase, and *γ*-GT in *E. sinensis*, suggesting that CPH improved the nutrient digestibility (especially proteins and amino acids) of crabs. Supportively, both trypsin and pepsin play a major role in the digestion of dietary proteins [32], while both Na^+^/-K^+^-ATPase and *γ*-GT are closely involved in amino acid transport and metabolism [33]. Consistently, previous researches have confirmed that enzymatic digestion reduces the content of several antinutritional factors in plant proteins, like the trypsin inhibitor [34] in soybean meal and the pepsin inhibitor in cottonseed meal [35], thereby alleviating their inhibitions on the activities of intestinal enzymes.

In this research, the muscle total amino acid content of *E. sinensis* trended upward with the addition of CPH from 0.2% to 1.6%, but no significant effect among different groups. The reason is presumed to be that the synthesis of muscle in decapod crustacean is strictly controlled by the genome [36–38]. In contrast, CPH supplement remarkably increased the MY and muscle crude protein content. This may be ascribed to the enhanced content of small peptides and free amino acids in CPH, both of which have been reported to promote protein synthesis in aquatic species [39]. Generally, protein synthesis and catabolism occur in parallel in the body of animals. When protein synthesis is greater than its catabolism, a tendency for muscle growth is usually observed [40]. To further unveil the potential mechanisms underlying this beneficial result, the relative expressions of MSTN and EsTGFBRI were measured. In this study, dietary additions of CPH remarkably decreased the transcriptions of both MSTN and EsTGFBRI. This was in conformity with the findings obtained in both MY and muscle protein content. This result is reasonable, since MSTN can inhibit the muscle growth by decreasing protein synthesis while increasing protein degradation [36], and that EsTGFBRI has been shown to negatively regulate the muscle growth in *E*. *sinensis* [23].

Crustaceans have rigid exoskeletons that require the physiological process of molting for growth [41]. When the condition factor of *E. sinensis* reaches over 60%, the current exoskeleton is not sufficient for the continued growth and development. The molting activity is then initiated in order to move to the next stage of growth [7]. In this study, the addition of CPH markedly improved the molting performance of *E. sinensis*. As described in the growth performance section, CPH is rich in free amino acids and small peptides, which could promote the nutrient deposition and muscle growth in aquatic species. Therefore, the CPH group initiated the molting activity earlier than the control group in order to satisfy the need for rapid growth to enter the next growth stage. In addition, the increase of hydrolysate content in CPH also helped to increase the hemolymph osmotic pressure of *E. sinensis* [22], as it contributes to the physiological process of muscle contraction in crustaceans, when they detach from the old exoskeleton [22]. To further investigate the potential mechanisms, the concentrations and transcriptions of molting-related hormones and genes were measured. The findings suggested that CPH increased the concentrations of both ecdysone and MF as well as the transcriptions of EcR, RXR, and CP cbm, but decreased the transcription of MIH. Supportively, ecdysone and MF are two important hormones that promote the molting activity in crustaceans [42]. Ecdysone is a steroid hormone that regulates the molting behavior by participating in several activities such as the dissolution and production of chitin and cuticle [41]. Meanwhile, MF, a precursor of juvenile hormone, also has a stimulating effect on the molting activity in crustaceans [43]. In addition, EcR forms a dimer with RXR, and binds to the ecdysone response element to regulate the biological activity of ecdysone [44]. Unlikely, MIH exerts a negative feedback on the molting activity by inhibiting the mTOR signaling and the secretion of Y-organ ecdysone [45]. Moreover, supplementation of 0.8% CPH in diet markedly increased the transcription of CP cbm, when compared with the control group. This again reinforced the fact that CPH could enhance the molting performance of *E. sinensis*. Generally, a complete molting activity in crustaceans is also dependent on the formation of new epidermis, which presupposes the creation of new cuticle (underlying the hard exoskeleton) that will harden the exoskeleton at the next growth stage [46]. CP cbm has an important function in epidermis formation and its expression positively correlated with the molting rate [24]. Together, these results further validated that CPH improved the growth performance of *E. sinensis* by promoting the molting activity.

## 5. Conclusion

**Figure 5:**
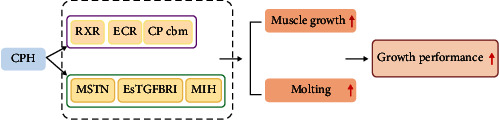
Graphical summary of CPH to improve the growth performance by improving the muscle growth and molt-related genes in the *E. sinensis*. CPH, cottonseed meal protein hydrolysate; RXR, retinoid X receptor; EcR, ecdysteroid receptor; CP cbm, cuticle protein cbm; MSTN, myostatin; EsTGFBRI, transforming growth factor-*β* type I receptor; MIH, molt-inhibiting hormone.

In conclusion, dietary supplementation of CPH at 0.4%–3.2% significantly improved the growth performance of *E. sinensis*. This beneficial effect was achieved partly by promoting the muscle growth and molting performance, as it was evidenced by the increased muscle yield, muscle protein content, the molting ratio, and the concentrations/transcriptions of molting-related hormones and genes (Figure 5). In practical farming, the amount of CPH should be adjusted according to the farming requirements (e.g., the pursuit of SR, or weight growth and MY, or FCR) to improve the economic efficiency.

## Figures and Tables

**Table 1:** The nutritive peculiarity of cottonseed meal (CM) and cottonseed meal protein hydrolysate (CPH).

Nutritive peculiarity	CM	CPH
Proximate composition (g/kg, dry-matter basis)		
Ash	64.5	68.7
Crude protein	540.0	595.4
Ether extract	7.4	9.7
Gross energy (MJ/kg)	11.1	12.3
Free gossypol (mg/kg)	864.7	839.2
Protein profile		
Soluble protein	10.4	23.4
Amino acid (74–180 Da)	3.2	5.1
Small peptides (180–1983 Da)	1.9	13.4
Proteins (1,893–21,828 Da)	5.2	4.9
Essential amino acids (g/kg, dry-matter basis)		
Arginine	63.3	62.8
Histidine	14.0	14.5
Lysine	19.8	20.0
Isoleucine	15.1	15.0
Leucine	29.6	29.6
Methionine	8.4	8.9
Phenylalanine	28.1	30.1
Tryptophane	14.5	15.0
Threonine	16.9	16.7
Valine	21.7	21.8

The data were collected from previous literatures [10, 17, 18].

**Table 2:** Formulation and proximate composition of the experimental diets.

Ingredients (%)	CPH0	CPH0.2	CPH0.4	CPH0.8	CPH1.6	CPH3.2
Fish meal	30.00	30.00	30.00	30.00	30.00	30.00
Blood meal	4.00	4.00	4.00	4.00	4.00	4.00
Soybean meal (defatted)	10.00	10.00	10.00	10.00	10.00	10.00
Cotton meal	4.00	3.78	3.56	3.12	2.24	0.47
CPH	0	0.20	0.40	0.80	1.60	3.20
Peanut meal	18.81	18.81	18.81	18.81	18.81	18.81
Rapeseed meal	2.00	2.00	2.00	2.00	2.00	2.00
*α*-Starch	20.93	20.95	20.97	21.01	21.10	21.26
Soybean oil	3.55	3.55	3.55	3.55	3.55	3.55
Fish oil	1.00	1.00	1.00	1.00	1.00	1.00
Ca(H_2_PO_4_)_2_	1.50	1.50	1.50	1.50	1.50	1.50
Zeolite powder	0.9	0.9	0.9	0.9	0.9	0.9
Premix^a^	1.00	1.00	1.00	1.00	1.00	1.00
Mixture^b^	2.30	2.30	2.30	2.30	2.30	2.30
Proximate composition (g/kg, dry-matter basis)						
Dry matter	89.46	89.55	89.36	89.94	89.79	90.36
Crude protein	39.78	39.85	39.91	39.87	39.79	39.78
Crude lipid	7.51	7.49	7.48	7.50	7.49	7.46

^a^Premix supplied the following minerals (g/kg) and vitamins (IU or mg/kg) per kilogram: CuSO_4_·5H_2_O, 2.0 g; FeSO_4_·7H_2_O, 25 g; ZnSO_4_ · 7H_2_O, 22 g; MnSO_4_·4H_2_O, 7 g; Na_2_SeO_3_, 0.04 g; KI, 0.026 g; CoCl_2_·6H_2_O, 0.1 g; vitamin A, 900,000 IU; vitamin D, 200,000 IU; vitamin E, 4,500 mg; vitamin K_3_, 220 mg; vitamin B_1_, 320 mg; vitamin B_2_, 1,090 mg; vitamin B_5_, 2,000 mg; vitamin B_6_, 500 mg; vitamin B_12_, 1.6 mg; vitamin C, 10,000 mg; pantothenate, 1,000 mg; folic acid, 165 mg; choline, 60,000 mg; biotin, 100 mg; and *Myo*-inositol 15,000 mg. ^b^Mixture includes the following ingredients (%): choline chloride 4.21%; antioxidants 1.26%; mildew-proof agent 2.09%; salt 21.03%; lvkangyuan 63.15%; and biostimep 8.26%.CPH, cottonseed meal protein hydrolysate; CPH0 to CPH3.2, dietary cotton meal replacement by 0%, 0.2%, 0.4%, 0.8%, 1.6%, and 3.2% of CPH.

**Table 3:** Nucleotide sequences of the primers used in real-time PCR.

Gene	Forward (5′–3′)	Reverse (5′–3′)	Accession number or reference
*MSTN*	AATGGCGAGTGTCCCTTCCTG	GTGGTCGTGGTCGTAGTAGAGC	Yue et al. [22]
*EsTGFBRI*	GGGACGGACATGTAGGAC	TCCTCACGCTCATTGGCT	Tian et al. [23]
*EcR*	CTCCCGGGTGCCATATTACC	TGCTACACGGCACATTCACT	KF469223.1
*RXR*	ACCCTGTGCTAACCCTCTGA	TGCTCACCACATCCTGCTTT	MK604180.1
*MIH*	TTTAGCTCCGTTCACGCCTT	TGGAGAACCCAGGAAAGCAC	DQ341280.1
*Cc*	CAACGACGACATCAAGCTGC	CATAACCGTGAGCAATGGCG	JX162648.1
*CP cbm*	CTGTTGCCTCA TCCCGAAAA	ATTGTACTCCCAGTTGCATGTCAC	Huang et al. [24]
*S27*	GGTCGATGACAATGGCAAGA	CCACAGTACTGGCGGTCAAA	Huang et al. [25]

MSTN, myostatin; EsTGFBRI, transforming growth factor-*β* type I receptor; EcR, ecdysteroid receptor; RXR, retinoid X receptor; MIH, molt-inhibiting hormone; Cc, cryptocyanin; CP cbm, cuticle protein cbm; S27, ubiquitin/ribosomal S27 fusion protein.

**Table 4:** Effects of dietary cottonseed meal protein hydrolysate levels on the growth performance of *E. sinensis*.

Group	CPH0	CPH0.2	CPH0.4	CPH0.8	CPH1.6	CPH3.2	Polynomial contrasts	
							Linear	Quadratic
IBW (g)	37.33 ± 0.67	37.40 ± 0.23	37.33 ± 0.24	37.33 ± 0.07	37.53 ± 0.35	37.00 ± 0.00	ns	ns
FBW (g)	60.50 ± 1.18^c^	61.73 ± 0.59^bc^	62.04 ± 0.21^bc^	63.24 ± 0.31^abc^	64.27 ± 0.38^ab^	65.18 ± 0.60^a^	0.00	0.00
WG^1^ (%)	61.97 ± 3.11^c^	65.02 ± 1.46^bc^	66.80 ± 0.72^bc^	69.78 ± 0.89^ab^	70.99 ± 1.30^ab^	76.16 ± 1.63^a^	0.00	0.00
SR^2^ (%)	67.50 ± 4.79^b^	65.00 ± 2.89^b^	82.50 ± 2.50^a^	65.00 ± 2.89^b^	72.50 ± 2.50^ab^	67.50 ± 2.50^b^	ns	ns
FCR^3^	2.07 ± 0.34^a^	1.94 ± 0.05^ab^	1.88 ± 0.07^ab^	1.96 ± 0.05^ab^	1.82 ± 0.05^b^	1.74 ± 0.05^b^	0.00	0.01
FI^4^ (g)	64.44 ± 8.01	78.29 ± 6.49	65.67 ± 10.23	83.00 ± 8.54	69.29 ± 4.10	72.14 ± 4.86	ns	ns
BPG^5^ (g)	3.41 ± 0.14^d^	3.51 ± 0.06^cd^	3.78 ± 0.02^c^	4.22 ± 0.04^b^	4.41 ± 0.07^ab^	4.64 ± 0.08^a^	0.00	0.00
APU^6^ (%)	6.83 ± 0.25^d^	7.32 ± 0.17^cd^	8.14 ± 0.32^c^	8.56 ± 0.20^bc^	9.49 ± 0.38^ab^	10.22 ± 0.30^a^	0.00	0.00

Values are means ± SEM of four replicates. Means in the same row with different superscript letters are significantly different (*P* < 0.05). ns, not significant. CPH, cottonseed meal protein hydrolysate; CPH0 to CPH3.2, dietary cotton meal replacement by 0%, 0.2%, 0.4%, 0.8%, 1.6%, and 3.2% of CPH. IBW, initial body weight; FBW, final body weight; WG, weight gain; SR, survival rate; FCR, feed conversion ratio; FI, feed intake; BPG, body protein gain; APU, apparent protein utilization. Calculations were carried out using the following formulas: ^1^WG (%) = 100 × (final body weight–initial body weight)/initial body weight; ^2^SR (%) = 100 × final survival crab number/initial crab number; ^3^FCR = total diet fed/total weight gain; ^4^FI = total diet fed/crab number; ^5^BPG (g) = final amount of dry body protein–initial amount of dry body protein; ^6^APU (%) = 100 × BPG/dry protein intake; SEM, standard error of mean.

**Table 5:** Effects of dietary cottonseed meal protein hydrolysate levels on the muscle composition (%, wet weight) of *E. sinensis*.

Group	CPH0	CPH0.2	CPH0.4	CPH0.8	CPH1.6	CPH3.2	Polynomial contrasts	
							Linear	Quadratic
Moisture (%)	79.42 ± 0.03^ab^	79.56 ± 0.21^a^	79.27 ± 0.10^ab^	78.98 ± 0.11^ab^	78.51 ± 0.39^bc^	77.94 ± 0.14^c^	0.00	0.00
Crude protein (%)	16.58 ± 0.17^c^	16.49 ± 0.14^c^	16.72 ± 0.09^c^	16.94 ± 0.11^bc^	17.51 ± 0.20^ab^	17.71 ± 0.07^a^	0.00	0.00
Crude lipid (%)	1.25 ± 0.03	1.25 ± 0.01	1.29 ± 0.03	1.24 ± 0.05	1.27 ± 0.01	1.26 ± 0.01	ns	ns
Crude ash (%)	1.52 ± 0.01	1.44 ± 0.04	1.50 ± 0.02	1.46 ± 0.01	1.51 ± 0.04	1.45 ± 0.03	ns	ns

Values are means ± SEM of four replicates. Means in the same row with different superscript letters are significantly different (*P* < 0.05). ns, not significant. CPH, cottonseed meal protein hydrolysate; CPH0 to CPH3.2, dietary cotton meal replacement by 0%, 0.2%, 0.4%, 0.8%, 1.6%, and 3.2% of CPH; SEM, standard error of mean.

**Table 6:** Effect of dietary cottonseed meal protein hydrolysate levels on the muscle amino acid composition (mg/g tissue wet weight) in the *E. sinensis*.

Amino acids	CPH0	CPH0.2	CPH0.4	CPH0.8	CPH1.6	CPH3.2	Polynomial contrasts	
							Linear	Quadratic
EAA			
Threonine	7.73 ± 0.10	7.60 ± 0.08	7.54 ± 0.06	7.61 ± 0.14	7.83 ± 0.06	7.59 ± 0.11	ns	ns
Valine	7.70 ± 0.12	7.77 ± 0.13	7.65 ± 0.08	7.76 ± 0.14	7.90 ± 0.04	7.74 ± 0.16	ns	ns
Methionine	4.75 ± 0.08	4.61 ± 0.10	4.89 ± 0.09	4.63 ± 0.01	4.72 ± 0.14	4.55 ± 0.07	ns	ns
Isoleucine	6.28 ± 0.11	6.16 ± 0.18	6.01 ± 0.18	6.02 ± 0.21	5.94 ± 0.07	6.09 ± 0.07	ns	ns
Leucine	11.20 ± 0.05	11.17 ± 0.26	11.22 ± 0.20	10.89 ± 0.17	11.29 ± 0.07	11.08 ± 0.23	ns	ns
Phenylalanine	6.67 ± 0.05	7.19 ± 0.21	7.07 ± 0.27	6.76 ± 0.24	6.98 ± 0.13	7.35 ± 0.15	ns	ns
Lysine	11.18 ± 0.44	11.68 ± 0.41	11.10 ± 0.43	11.80 ± 0.24	11.36 ± 0.22	11.49 ± 0.15	ns	ns
Histidine	4.19 ± 0.27	3.89 ± 0.18	4.15 ± 0.15	3.97 ± 0.29	4.33 ± 0.13	3.90 ± 0.18	ns	ns
Arginine	16.29 ± 0.39	16.24 ± 0.36	16.57 ± 0.09	16.58 ± 0.26	17.08 ± 0.50	16.88 ± 0.23	ns	ns
*Σ* EAA	75.98 ± 1.11	76.31 ± 0.29	76.22 ± 0.60	76.02 ± 0.45	77.87 ± 0.58	76.56 ± 0.76	ns	ns
NEAA								
Asparagine	15.45 ± 0.08	15.44 ± 0.10	15.38 ± 0.14	15.41 ± 0.06	15.48 ± 0.01	15.55 ± 0.03	ns	ns
Serine	7.33 ± 0.17	6.63 ± 0.15	7.14 ± 0.15	6.66 ± 0.41	6.86 ± 0.20	6.84 ± 0.21	ns	ns
Glutamic acid	25.65 ± 0.49	25.40 ± 0.18	25.01 ± 0.26	26.41 ± 0.26	25.72 ± 0.61	25.73 ± 0.42	ns	ns
Glycine	10.45 ± 0.07	10.20 ± 0.21	10.48 ± 0.16	10.43 ± 0.37	10.47 ± 0.08	10.35 ± 0.15	ns	ns
Alanine	12.20 ± 0.15	11.92 ± 0.15	12.37 ± 0.11	12.23 ± 0.27	12.65 ± 0.14	12.51 ± 0.16	ns	ns
Cysteine	2.00 ± 0.14	1.88 ± 0.04	1.83 ± 0.13	2.13 ± 0.09	1.87 ± 0.08	1.82 ± 0.20	ns	ns
Tyrosine	6.23 ± 0.18	6.61 ± 0.33	6.73 ± 0.12	6.94 ± 0.25	6.84 ± 0.15	6.68 ± 0.25	ns	ns
Proline	7.51 ± 0.08	7.25 ± 0.14	7.25 ± 0.17	7.05 ± 0.07	7.43 ± 0.14	7.51 ± 0.06	ns	ns
*Σ* NEAA	86.82 ± 0.91	85.33 ± 0.78	86.20 ± 0.67	87.26 ± 0.59	86.98 ± 0.79	86.65 ± 0.31	ns	ns
*Σ* AA	162.80 ± 2.00	161.64 ± 0.85	162.42 ± 1.17	163.28 ± 0.86	165.54 ± 0.25	163.15 ± 0.25	ns	ns

Values are means ± SEM of three replicates. Means in the same row with different superscript letters are significantly different (*P* < 0.05). ns, not significant. CPH, cottonseed meal protein hydrolysate; CPH0 to CPH3.2, dietary cotton meal replacement by 0%, 0.2%, 0.4%, 0.8%, 1.6%, and 3.2% of CPH; *Σ* EAA, total essential amino acids; *Σ* NEAA, total nonessential amino acids; *Σ* AA, total amino acids; SEM, standard error of mean.

## Data Availability

The data of this research can be obtained from the corresponding author under reasonable requirements.
